# The EPA oxylipin, 12-HEPE, directly regulates human platelet activity

**DOI:** 10.1016/j.jlr.2025.100807

**Published:** 2025-04-16

**Authors:** Krista Goerger, Livia Stanger, Andrew Rickenberg, Anthony Nguyễn, Taekyu Lee, Theodore R. Holman, Michael Holinstat

**Affiliations:** 1 Department of Pharmacology, University of Michigan Medical School, Ann Arbor, MI, USA; 2 Department of Chemistry and Biochemistry, University of California Santa Cruz, Santa Cruz, CA, USA; 3 Department of Internal Medicine, University of Michigan Medical School, Ann Arbor, MI, USA

**Keywords:** platelets, omega-3 fatty acids, lipoxygenase, oxidized lipids, cyclooxygenase, eicosapentaenoic acid, cardiovascular disease, thrombosis, fish oil

## Abstract

Arterial thrombosis, driven by platelet hyperactivity, is the underlying pathophysiology of most major cardiovascular events. Dietary fish oil supplementation containing ω-3 polyunsaturated fatty acids (PUFAs) elicits cardiovascular protection in at-risk patients. Studies have attributed the cardiovascular benefits of ω-3 PUFAs to eicosapentaenoic acid (EPA), the primary ω-3 PUFA present in fish oil supplements. However, the role of EPA in platelet activation remains unclear. This study aimed to evaluate whether the cardiovascular protection observed in individuals taking dietary supplements containing EPA is achieved by altering platelet function. Additionally, we investigated whether these effects are mediated through the 12-lipoxygenase (12-LOX)-derived oxidized lipid (oxylipin) metabolite of EPA, 12(S)-hydroxy-5Z,8Z,10E,14Z,17Z-eicosapentaenoic acid (12-HEPE). Human whole blood, platelet-rich plasma, and washed platelets were treated with EPA or 12-HEPE to assess their ability to regulate platelet activity. Both EPA and 12-HEPE inhibited agonist-stimulated platelet aggregation, and 12-HEPE was found to be the primary oxylipin produced by platelets in the presence of EPA. Furthermore, 12-HEPE more potently attenuated dense granule secretion, α-granule secretion, and integrin α_IIb_β_3_ activation, in comparison to EPA. Interestingly, while EPA delayed thrombin-induced clot retraction and reduced platelet adhesion under flow, 12-HEPE did not affect these processes. Both EPA and 12-HEPE attenuated ex vivo thrombus formation; however, the same inhibitory concentrations did not alter coagulation parameters in thromboelastography. This study demonstrates that EPA and its 12-LOX metabolite, 12-HEPE, effectively inhibit platelet activation. These findings suggest the antiplatelet effects of EPA are regulated, in part, through 12-HEPE, advancing our understanding of the cardiovascular benefits of EPA.

Platelets play a critical role in the hemostatic response following vascular injury, mediating arterial clot formation to reduce blood loss (1). However, in pathological conditions, abnormal platelet activation can lead to the formation of an occlusive thrombus, obstructing blood flow to major organs and causing cardiovascular complications such as myocardial infarction and stroke (2). Increased dietary intake of ω-3 polyunsaturated fatty acids (PUFAs) has long been used to increase cardiovascular protection (3, 4, 5, 6, 7). Since 2002, the American Heart Association has recommended increased consumption of ω-3 PUFAs, either through food or supplementation (8), and continues to recommend ω-3 PUFA supplementation for patients with increased risk of cardiovascular disease and stroke (9).

Fish oil supplements are the primary nonprescription source of ω-3 PUFAs and are the third most common over-the-counter supplement in the United States (10). Fish oil supplements have significant cardiovascular benefits, including lowering triglycerides (5, 11) and decreasing platelet activity (12), and provide high levels of the ω-3 PUFAs, docosahexaenoic acid (DHA), and eicosapentaenoic acid (EPA) (13). Several studies have attributed the cardiovascular benefits of fish oil supplements to EPA, as EPA is the primary component of fish oil supplements (14), and supplementation with EPA alone reduces the risk for cardiovascular events (15, 16). Further, the recent success of the REDUCE-IT trial demonstrates supplementation with icosapent ethyl (IPE), a highly pure esterified form of EPA, reduces the risk for major cardiovascular events in patients at risk for CVD (17). However, despite the wide use of dietary supplements with high levels of EPA, the exact mechanisms through which EPA elicits cardiovascular protection are unknown.

PUFAs can regulate platelet function through their bioactive metabolites, or oxidized lipids (oxylipins), generated by oxygenase enzymes (18). EPA is a known substrate for the two major oxygenases in the platelet, cyclooxygenase (COX)-1 and 12-lipoxygenase (LOX) (19, 20). 12-LOX plays a key role in platelet reactivity, and the antiplatelet effects of several PUFAs are observed through their 12-LOX oxylipins (20, 21, 22, 23). The metabolism of EPA by 12-LOX is known to yield 12(S)-hydroperoxy-5Z,8Z,10E,14Z,17Z-eicosapentaenoic acid (12-HpEPE), which is quickly reduced to 12(S)-hydroxy-5Z,8Z,10E,14Z,17Z-eicosapentaenoic acid (12-HEPE) in platelets (20). However, the role of 12-LOX in mediating the effects of EPA has not been investigated.

We hypothesize that EPA inhibits platelet activation, in part, through its 12-LOX oxylipin, 12-HEPE, contributing to the vascular protective effects realized in individuals taking dietary supplements containing high levels of EPA. We set out to determine the effects of EPA and 12-HEPE on human platelet activation and thrombus formation. In this study, we demonstrate that 12-HEPE is the primary oxylipin produced from EPA, and both EPA and 12-HEPE inhibit platelet activation. For the first time, we provide evidence supporting the role of 12-LOX in regulating the antithrombotic effects of EPA and uncover a potential mechanism through which EPA reduces the risk for major cardiovascular events.

## Materials and Methods

### Production and isolation of 12-HEPE

The synthesis of 12-HEPE was performed as previously described (24, 25). Briefly, 12-HpEPE was synthesized by reaction of EPA (25–50 μM) with human 12(S)-lipoxygenase. The hydroperoxide product, 12-HpEPE, was reduced to the alcohol, 12-HEPE with trimethylphosphite, and then purified by HPLC using a C18 HAISIL 250 × 10 mm semi-prep column isocratically in a mobile phase containing 54.5:44.5:1 mixture of acetonitrile, water, and formic acid, respectively. The purity was determined to be greater than 95% via analytical HPLC and MS analysis (vide infra).

### Preparation of washed human platelets

All research involving human subjects was carried out in accordance with the Declaration of Helsinki. The University of Michigan Institutional Review Board approved all experiments involving human participants (approval number: HUM00100677). A signed consent form was obtained from self-reported healthy donors prior to blood draws. Whole blood was collected via venipuncture into vacutainers containing sodium citrate (3.2%; Greiner Bio-One). Platelets were isolated from whole blood via serial centrifugation. Whole blood was centrifuged at 200 *g* for 15 min to isolate platelet-rich plasma (PRP). To pellet the platelets, PRP was treated with acid citrate dextrose (2.5% sodium citrate tribasic, 1.5% citric acid, and 2.0% D-glucose) and apyrase (0.2 U/ml) and centrifuged at 2000 *g* for 10 min. Platelets were resuspended in Tyrode's buffer (10 mM HEPES, 12 mM sodium bicarbonate, 127 mM sodium chloride, 5 mM potassium chloride, 0.4 mM monosodium phosphate, 1 mM magnesium chloride, and 180 mM D-glucose) to a physiological concentration of 3.0 × 10^8^ platelets/ml determined by a complete blood cell counter (Hemavet 950FS; Drew Scientific) (21, 26).

### Quantification of oxylipins via mass spectrometry

To collect the platelet releasate, washed human platelets were stimulated with 1 μg/ml collagen (Chrono-Log) or 0.5 nM thrombin (Enzyme Research Labs, South Bend, IN) following incubation with vehicle (DMSO) or 10 μM EPA for 10 min at 37°C. After 10 min of stimulation with the agonist, the platelet releasate was collected from 3.0 × 10^8^ platelets following centrifugation at 2000 *g* for 2 min (23). To collect serum, whole blood was incubated with vehicle (DMSO) or 500 μM EPA for 25 min and centrifuged at 2,000 *g* for 10 min. The samples were resuspended in water, extracted three times with dichloromethane, reduced with trimethyl phosphite, and evaporated under a stream of nitrogen gas. Reactions were analyzed via liquid chromatography-mass spectrometry (LC-MS/MS). The chromatography system was coupled to a Thermo-Electron LTQ LC-MS/MS for mass analysis. All analyses were performed in negative ionization mode at the normal resolution setting. Mass spectrometry was performed in a targeted manner with a negative ion mass list containing the following m/z ratios ± 0.5: 317.5/115 (5(S)-hydroxyeicosapentaenoic acid; 5-HEPE), 317.5/179 (12-HEPE), 317.5/219 (15(S)-hydroxyeicosapentaenoic acid; 15-HEPE), 319.5/115 (5(S)-hydroxyeicosatetranoic acid; 5-HETE), 319.5/219 (15(S)-hydroxyeicosatetranoic acid; 15-HETE), and 367.5/169 (thromboxane B_3_; TxB_3_). Because thromboxane A_3_ (TxA_3_) is a relatively unstable metabolite, the stable form TxB_3_ was measured as a surrogate for TxA_3_ formation in human platelets.

### Quantification of 12-HETE and TxB_2_ via ELISA

Washed human platelets were treated vehicle (DMSO) or 10 μM EPA for 10 min at 37°C and stimulated with 1 μg/ml collagen (Chrono-Log) or 0.5 nM thrombin (Enzyme Research Labs). After 10 min of stimulation, 3.0 × 10^8^ platelets were centrifuged at 2000 *g* for 2 min to collect the platelet releasate. To collect serum, whole blood was centrifuged at 2000 *g* for 10 min following a 25 min incubation with vehicle (DMSO) or 500 μM EPA. 12(S)-hydroxyeicosatetranoic acid (12-HETE) and thromboxane B_2_ (TxB_2_) were quantified via 12(S)-HETE and TxB_2_ ELISA according to the manufacturer's specifications (Cayman Chemical). Due to instability of thromboxane A_2_ (TxA_2_), its stable metabolite, TxB_2_, was measured as a surrogate for the TxA_2_ production in human platelets.

### Platelet aggregation

Washed human platelets at a physiological concentration (3.0 × 10^8^ platelets/ml) incubated with increasing concentrations of EPA (Nu-Chek Prep, Inc.), 12-HEPE, or an equivalent volume of vehicle (DMSO) for 10 min at 37°C. Platelets were stimulated with an EC_80_ of collagen (EC_80_ range: 0.125–1 μg/ml) or thrombin (EC_80_ range: 0.25–0.375 nM) (Enzyme Research Labs) and dose-response curves for EPA and 12-HEPE were attained. Platelet aggregation was measured via light transmission for 10 min at 37°C under stirring conditions (1,200 rpm) in a lumi-aggregometer (Model 700D; Chrono-log) (21).

### Dense granule secretion

Platelet ATP secretion was measured as a surrogate marker for dense granule secretion. Washed human platelets were treated with vehicle (DMSO), EPA, or 12-HEPE prior to stimulation with collagen (EC_80_ range: 0.125–0.5 μg/ml). CHRONO-LUME was added 1 min prior to stimulation, and luminescence was measured for 10 min at 37°C under stirring conditions (1,200 rpm) in a lumi-aggregometer (Model 700D; Chrono-log, Havertown, PA) (22).

### Flow cytometry

Washed human platelets were treated with vehicle (DMSO), EPA, or 12-HEPE for 10 min at 37°C. Collagen requires shear force to activate the platelet, so convulxin (Cayman Chemical, Ann Arbor, MI) was used as an agonist in the static setting of flow cytometry to directly activate the glycoprotein VI (GPVI) receptor in lieu of collagen. Treated platelets were stimulated with 12.5 ng/ml convulxin in the presence of FITC-conjugated antibody specific for the active conformation of integrin α_IIb_β_3_, PAC-1 (Biolegend), and APC/Cy7-conjugated CD62P antibody specific for P-selectin expressed on the surface of the platelet (BioLegend). Samples were incubated at 37°C for 10 min in the dark and were fixed with 2% paraformaldehyde. Fluorescence intensity was measured via flow cytometry (CytoFLEX, Beckman Coulter) (21).

### Clot retraction

PRP was isolated from citrated whole blood following centrifugation at 200 g for 15 min. Platelet count was adjusted to 3.0 × 10^8^ platelets/ml with platelet-poor plasma as determined by a complete blood cell counter (Hemavet 950FS; Drew Scientific). Following treatment with fatty acids (EPA or 12-HEPE) for 10 min at 37°C, clot retraction was initiated with 10 nM thrombin (Enzyme Research Labs). Pictures were taken of the clots every 15 min for 2 h. The size of the clot was determined via Image J software (20). Percent clot retraction was calculated and plotted over time, from which the time to 50% clot retraction was determined via nonlinear regression.

### Total thrombus formation analysis system

The total thrombus formation analysis system (T-TAS) is an automated microchip-based flow chamber system for assessment of thrombus formation under flow conditions. Human whole blood was anticoagulated with Benzylsulfonyl-D-Arg-Pro-4amidinobenzylamide (BAPA), a synthetic Factor Xa and IIa inhibitor (Diapharma), as recommended by the manufacturer. Blood was treated with vehicle (DMSO), EPA, or 12-HEPE for 10 min at 37°C. Whole blood was perfused at arterial shear (1,500 s^-1^) through a PL chip (Diapharma, West Chester, OH) containing capillary channels coated with collagen type I. Thrombus formation was assessed by monitoring the change in flow pressure (kPa) from capillary occlusion by adhered and aggregated platelets; the area under the flow pressure curve (AUC) was analyzed to quantify differences in thrombus formation (27, 28).

### Ex vivo microfluidic perfusion flow chamber

Microfluidic perfusion chamber slides (μ-slide VI 0.1, ibidi) were coated with 100 μg/ml collagen type I (Chrono-log) overnight at 4°C. Freshly drawn citrated whole blood was treated with EPA (0.01–1 mM), 12-HEPE (10–50 μM), or vehicle (DMSO) for 10 min at 37°C. Platelets were fluorescently labeled by incubating with 2 μM dihexyloxacarbocyanine iodide (DiOC_6_) (Thermo Fischer Scientific) for 5 min at 37°C. Stained whole blood was recalcified with 5 mM CaCl_2_ and immediately perfused at arterial shear (1800 s^-1^) through a coated microfluidic slide at 37°C (Harvard Apparatus). Platelet adhesion and accumulation were recorded in real time for 4 min under an inverted fluorescent microscope (20X objective, Zeiss Axio Observer Z1 Marianas; Zeiss Industrial Quality Solutions). Platelet accumulation was quantified by mean fluorescence intensity using Slidebook 7.0 (Intelligent Imaging Innovations) (21, 29).

### Thromboelastography

Whole blood drawn into vacutainers containing sodium citrate (3.2%; Greiner Bio-One, Monroe, NC) incubated with vehicle (DMSO), EPA, or 12-HEPE at 37°C for 10 min. CaCl_2_ (10 mM) was added to whole blood prior to analysis with a Haemoscope TEG 5000 Thrombelastograph Hemostasis Analyzer (Haemonetics Corp.). The viscoelastic properties of blood clot formation were analyzed, including time to formation of initial fibrin threads (reaction time), maximum clot strength (maximum amplitude), rate of clot formation (α-angle), time to specified clot strength (K time), lysis at 30 min (clot lysis), and estimated rate of clot formation (maximum rate of thrombin generation) (30, 31).

## Statistics

Prism 10 GraphPad software was used to analyze the data (GraphPad Software, La Jolla, CA). Data are presented as mean values ± standard error of the mean (SEM). Multiple statistical analyses were used in this study; the statistical test used in each assay is noted in the figure legend.

## Results

### EPA and 12-HEPE inhibit agonist-induced platelet aggregation

**Fig. 1:**
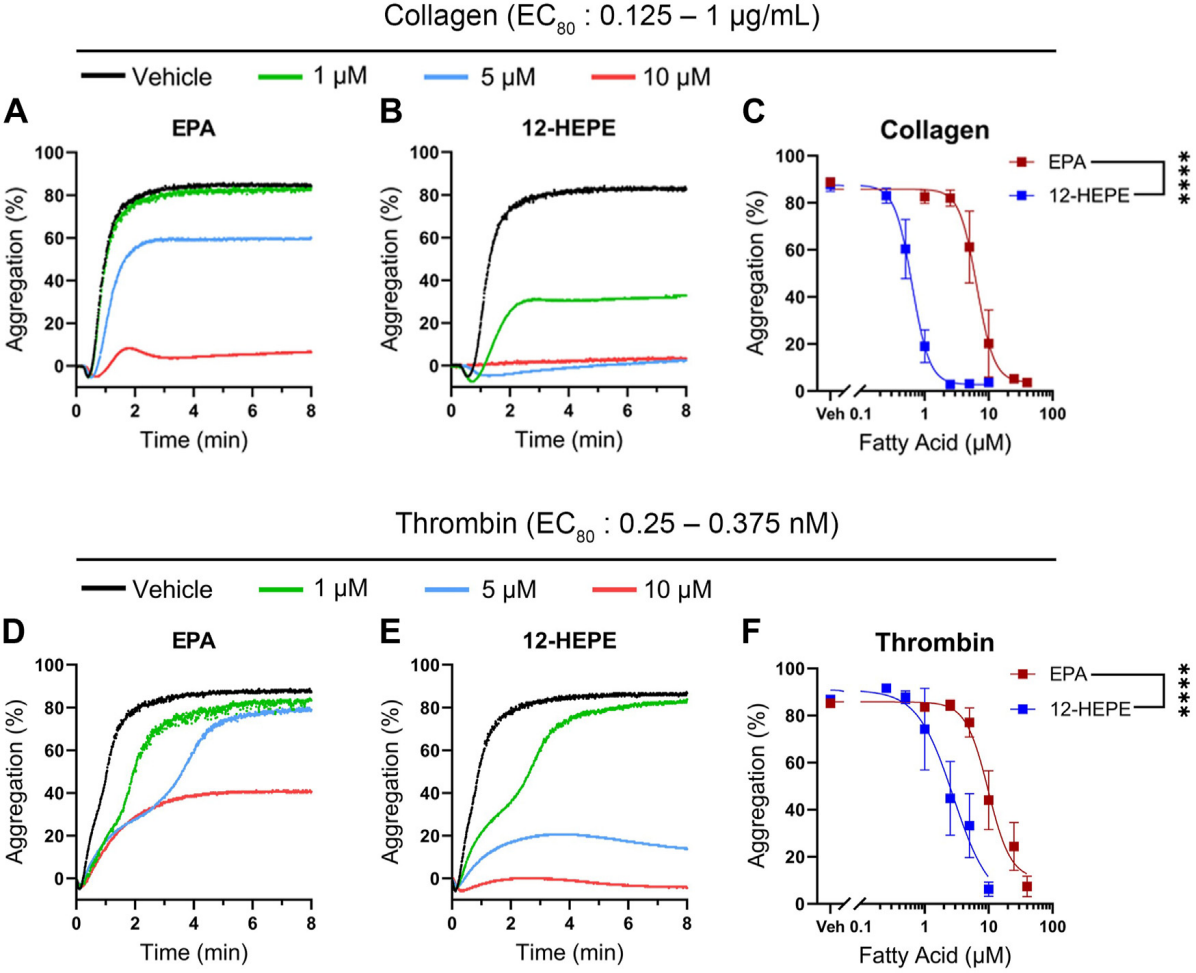
EPA and 12-HEPE inhibit platelet aggregation. Washed human platelets were treated with vehicle (DMSO) or increasing concentrations of EPA or 12-HEPE for 10 min followed by stimulation with an EC_80_ concentration of collagen (A–C) or thrombin (D–F). Aggregation was monitored for 10 min. Data are presented as representative trace curves (left and middle panels) and mean maximum percent aggregation ± SEM (right panels, n = 5). Four parameter nonlinear regression with extra sum-of-squares F test. Asterisks denote a statistical difference in EPA and 12-HEPE IC_50_ values. ∗∗∗∗*P* < 0.0001.

EPA and its 12-LOX-derived oxylipin, 12-HEPE, were assessed to determine their ability to regulate agonist-induced human platelet aggregation (Fig. 1). Washed human platelets were treated with increasing concentrations of EPA (1–40 μM) or 12-HEPE (0.25–10 μM) for 10 min prior to stimulation with collagen or thrombin, both of which are endogenous agonists in the blood vessel. The effective concentrations of collagen and thrombin reaching 80% aggregation (EC_80_) were used for each platelet sample to account for variations in human donor sensitivity to the agonists. EPA and its oxylipin, 12-HEPE, attenuated human platelet aggregation in a dose-dependent manner for both collagen (Fig. 1A–C) and thrombin (Fig. 1D–F). The IC_50_ values for EPA and 12-HEPE were 6.5 μM and 0.6 μM for collagen and 9.9 μM and 2.8 μM for thrombin, respectively. 12-HEPE exhibited increased potency with a 10-fold shift in IC_50_ compared to EPA when stimulated with collagen (Fig. 1C), and a 3.5-fold shift in IC_50_ when stimulated with thrombin (Fig. 1F).

### EPA induces oxylipin 12-HEPE production from platelets

**Fig. 2:**
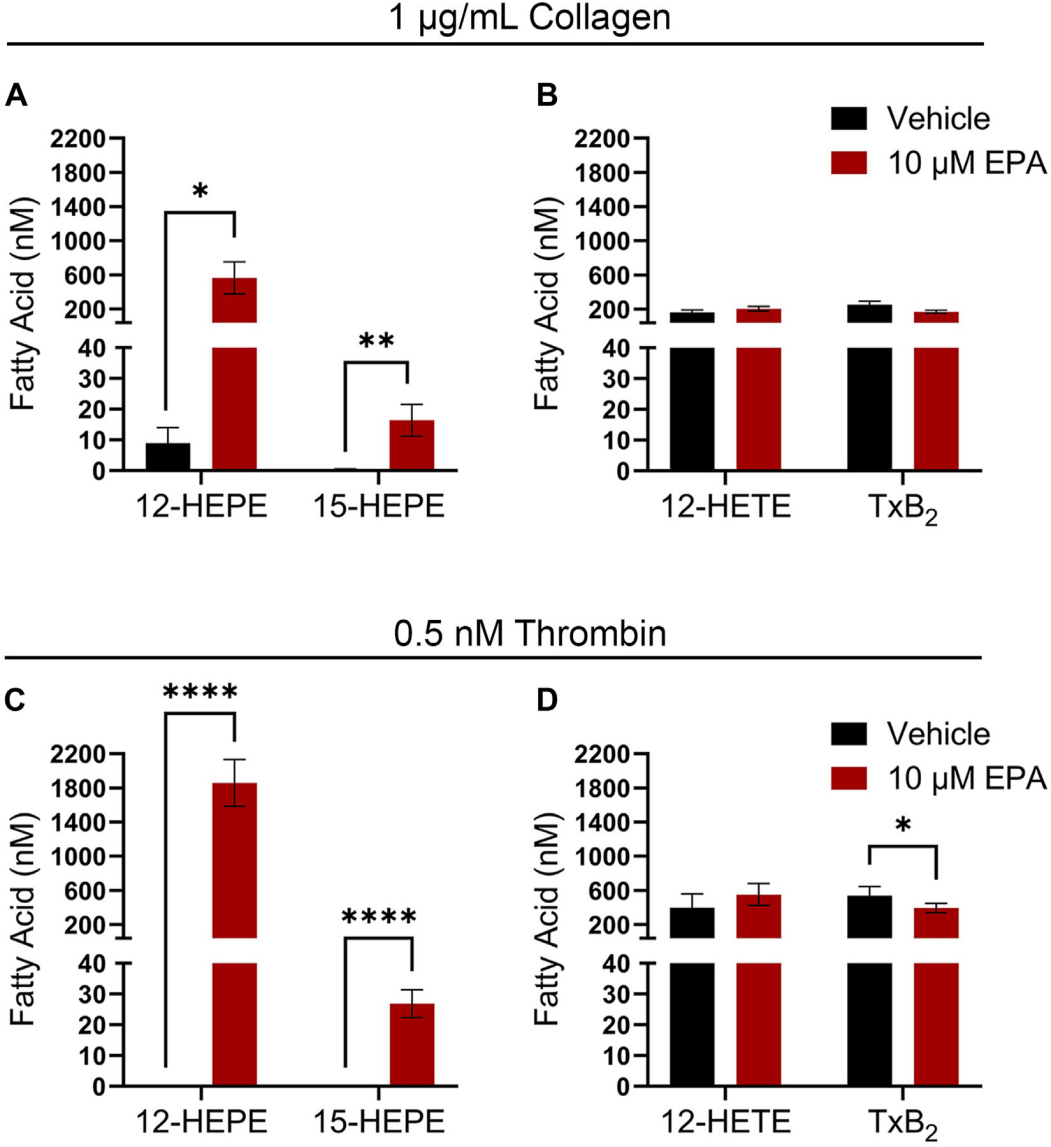
Exogenous EPA enhances platelet production of 12-HEPE. The 12-LOX and 15-LOX derived metabolites of EPA (A, C) and the 12-LOX and COX-1 derived metabolites from AA (B, D) were quantified in the platelet releasate collected from washed human platelets treated with vehicle (DMSO) or EPA (10 μM) and stimulated with collagen (1 μg/ml) or thrombin (0.5 nM). Data are presented as mean ± SEM (n = 4). Two-tailed paired *t* test; ∗*P* < 0.05, ∗∗*P* < 0.01, ∗∗∗∗*P* < 0.0001.

To determine if exogenous addition of EPA alters the production of platelet oxylipins, the lipid releasate from platelets stimulated with collagen or thrombin in the presence of vehicle or EPA (10 μM) were analyzed by LC/MS/MS and ELISA (Table 1 and Fig. 2). As expected, the amount of 12-HEPE, the EPA-derived 12-LOX oxylipin, was significantly higher in the EPA-treated group compared to the vehicle-treated group (Fig. 2A, C). The production of 15-HEPE was also increased in the presence of EPA, but at lower abundances compared to 12-HEPE (Fig. 2A, C). Interestingly, the EPA-derived COX-1 oxylipin (TxB_3_), which has been previously reported to be produced through COX-1 oxidation of the fatty acid (32), was undetected following incubation with EPA and stimulation with either collagen or thrombin (Table 1). Consistent with previous findings, the arachidonic acid (AA)-derived metabolites from 12-LOX (12-HETE) and COX-1 (TxB_2_) were produced at similar abundances (33), with thrombin resulting in higher concentrations of 12-HETE and TxB_2_ compared to collagen (Table 1 and Fig. 2B, D). The formation of 12-HETE was unaltered in platelets treated with EPA for both collagen and thrombin, while TxB_2_ is reduced in the presence of EPA following thrombin stimulation (Fig. 2B, D). Since platelets do not express 5-LOX (18), 5-LOX products were measured following addition of either AA or EPA (Table 1) to assess the presence of immune cells in the isolated platelet preparation. No 5-LOX products were identified in either condition.

**Table 1:** Platelet production of oxylipins in the presence of EPA

Oxygenase	PUFA	Oxylipin	Oxylipin Production (nM)			
			1 μg/ml Collagen		0.5 nM Thrombin	
			Vehicle	10 μM EPA	Vehicle	10 μM EPA
COX-1	AA	TxB_2_	(252.7 ± 42.8)	(169.0 ± 17.4)	(536.6 ± 107.1)	(394.4 ± 55.7)
	EPA	TxB_3_	0	0	0	0
12-LOX	AA	12-HETE	(162.0 ± 30.4)	(204.6 ± 27.0)	(396.8 ± 165.2)	(552.2 ± 128.7)
	EPA	12-HEPE	(9.0 ± 5.0)	(562.9 ± 188.3)	0	(2,310 ± 253.2)
15-LOX	AA	15-HETE	(6.4 ± 1.0)	(4.4 ± 1.2)	(1 ± 0.3)	(1 ± 0.2)
	EPA	15-HEPE	(0.5 ± 0.1)	(16.4 ± 5.2)	0	(35 ± 3.7)
5-LOX	AA	5-HETE	0	0	0	0
	EPA	5-HEPE	0	0	0	0

Platelet releasate was collected from washed human platelets incubated with vehicle (DMSO) or 10 μM EPA for 10 min and stimulated with 1 μg/ml collagen or 0.5 nM thrombin for 10 min. Oxylipins were analyzed via LC/MS/MS or ELISA are presented as mean ± SEM (n = 4).

### 12-HEPE attenuates granule secretion and integrin α_IIb_β_3_ activation

**Fig. 3:**
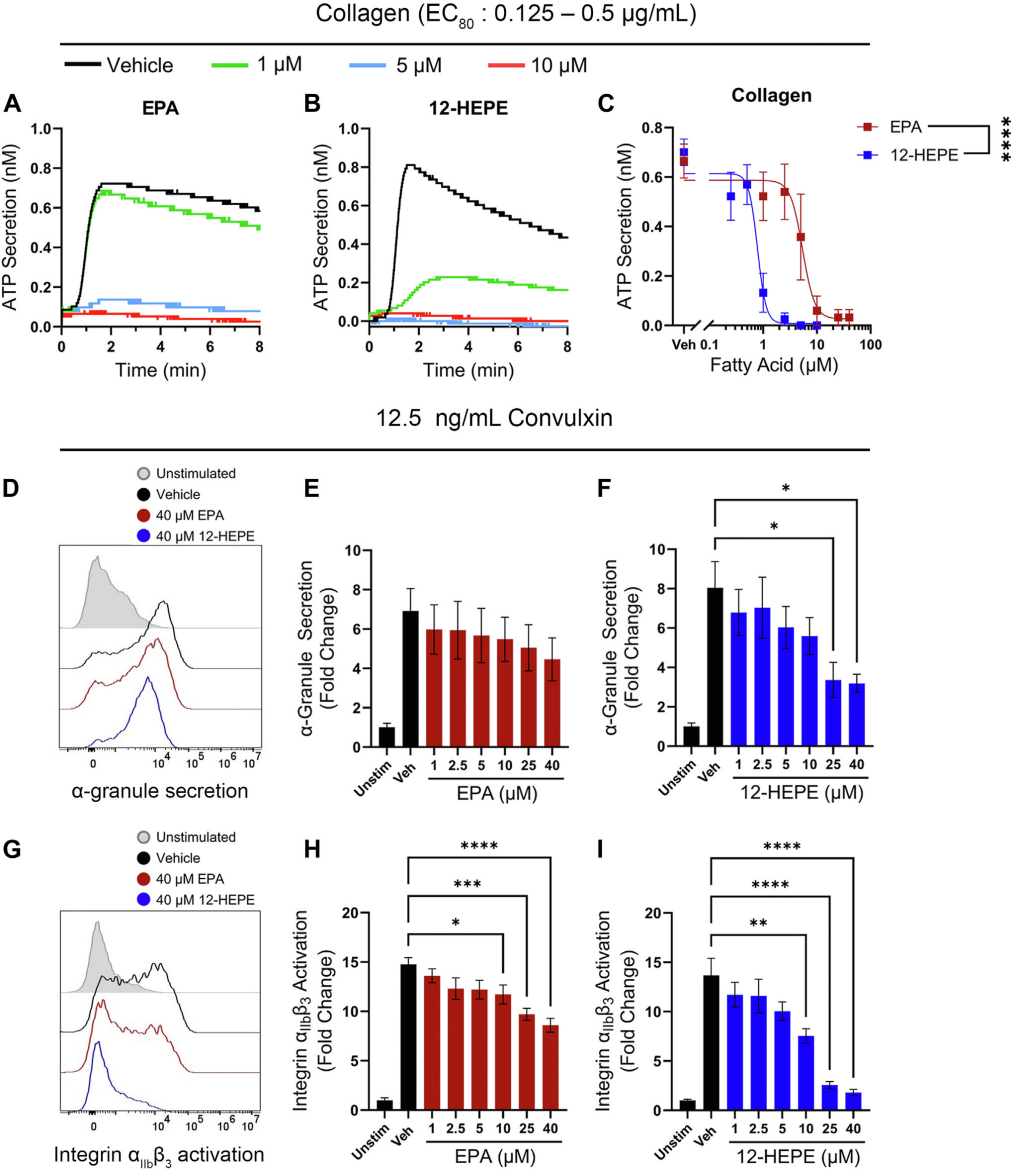
EPA and 12-HEPE regulate platelet granule secretion and integrin activation. ATP secretion was measured in washed human platelets treated with vehicle (DMSO), EPA, or 12-HEPE for 10 min prior to stimulation with the EC_80_ of collagen. Data are presented as representative trace curves (A, B) and mean ATP secretion ± SEM (C; n = 4). Four-parameter nonlinear regression with extra sum-of-squares F test. Asterisks denote a statistical difference in EPA and 12-HEPE IC_50_ values. α-granule secretion (D–F) and integrin α_IIb_β_3_ activation (G–I) were assessed in the presence of vehicle (DMSO), EPA, or 12-HEPE prior to stimulation with 12.5 ng/ml convulxin (n = 6). Data are presented as mean ± SEM. One-way ANOVA with Dunnett's multiple comparisons; ∗*P* < 0.05, ∗∗*P* < 0.01, ∗∗∗*P* < 0.001, ∗∗∗∗*P* < 0.0001.

Due to the observed inhibitory effects of EPA and 12-HEPE on platelet aggregation (Fig. 1), the ability of the fatty acids to alter granule secretion and integrin activation was assessed. To evaluate the effects on dense granule secretion, platelets were stimulated with an EC_80_ concentration of collagen in the presence of increasing concentrations of EPA or 12-HEPE. Both EPA and 12-HEPE were observed to attenuate ATP secretion, a marker of dense granule secretion, in a dose-dependent manner (Fig. 3A–C). Further, 12-HEPE exhibited an increased potency to inhibit granule secretion relative to EPA (Fig. 3C). To determine if EPA and 12-HEPE also altered α-granule secretion and integrin α_IIb_β_3_ activation, washed human platelets were stimulated with 12.5 ng/ml convulxin in the presence of EPA or 12-HEPE and analyzed via flow cytometry. Convulxin was used to stimulate the platelet GPVI receptor instead of collagen because collagen requires stirring or flowing conditions to expose the active form. An antibody that recognizes P-selectin expression on the surface of the platelet (CD62P) was used to analyze α-granule secretion and an antibody specific for the active conformation of integrin α_IIb_β_3_ (PAC-1) was used to analyze integrin α_IIb_β_3_ activation. 12-HEPE significantly inhibited agonist-induced α-granule expression on the surface of the platelet while EPA had no significant effect (Fig. 3D–F). A significant decrease in agonist-induced activation of α_IIb_β_3_ was observed at 10 μM for both EPA and 12-HEPE (Fig. 3G–I).

### EPA delays thrombin-induced clot retraction

**Fig. 4:**
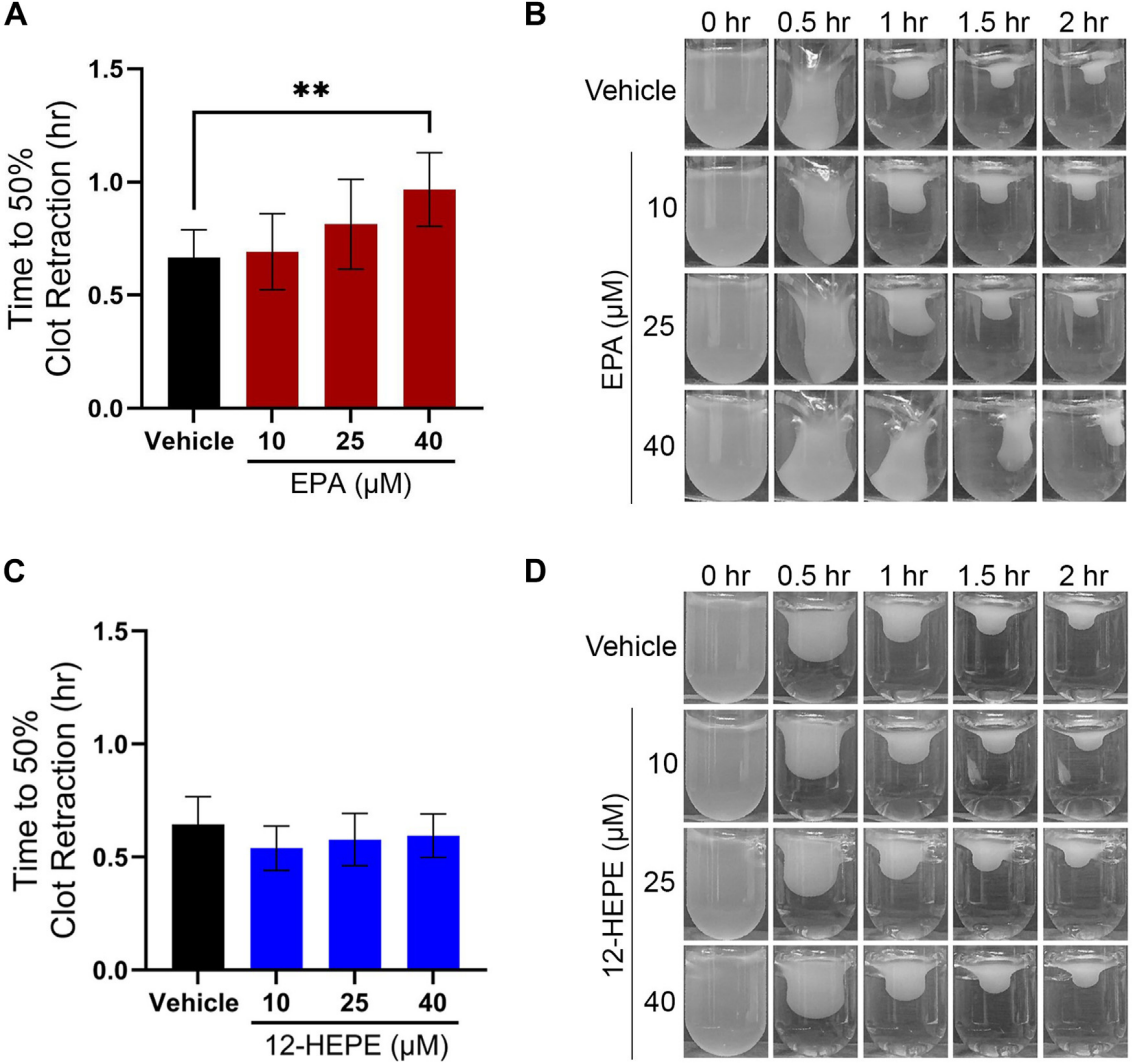
EPA delays thrombin-induced clot retraction. PRP was treated with the fatty acids EPA (A, B) or 12-HEPE (C, D) at 10, 25, and 40 μM, for 10 min followed by stimulation with 10 nM thrombin. Pictures were taken at several time points following stimulation (0, 0.5, 1, 1.5, and 2 h). The size of the clot was quantified using Image J. Data are presented as time to 50% clot retraction ± SEM (left panels; n = 3) and as representative images (right panels). One-way ANOVA with Dunnett's multiple comparisons; ∗∗*P* < 0.01.

To determine whether EPA or 12-HEPE alters the clot retraction process, or the platelet-dependent consolidation of the clot, PRP was treated with either fatty acid for 10 min. Thrombin (10 nM) was subsequently added to stimulate clot formation, and images were taken every 30 min for 2 h. The area of the clot was quantified at each time point via Image-J to determine the time to 50% clot retraction (Fig. 4). The vehicle treated PRP clotted within 1 h (20). Treatment with EPA significantly delayed clot retraction, but the clot was eventually able to retract, similar to vehicle-treated plasma 2 h after initial stimulation (Fig. 4A, B). The presence of 12-HEPE did not significantly alter the clot retraction process (Fig. 4C, D).

### EPA attenuates platelet adhesion and thrombus formation under flow

**Fig. 5:**
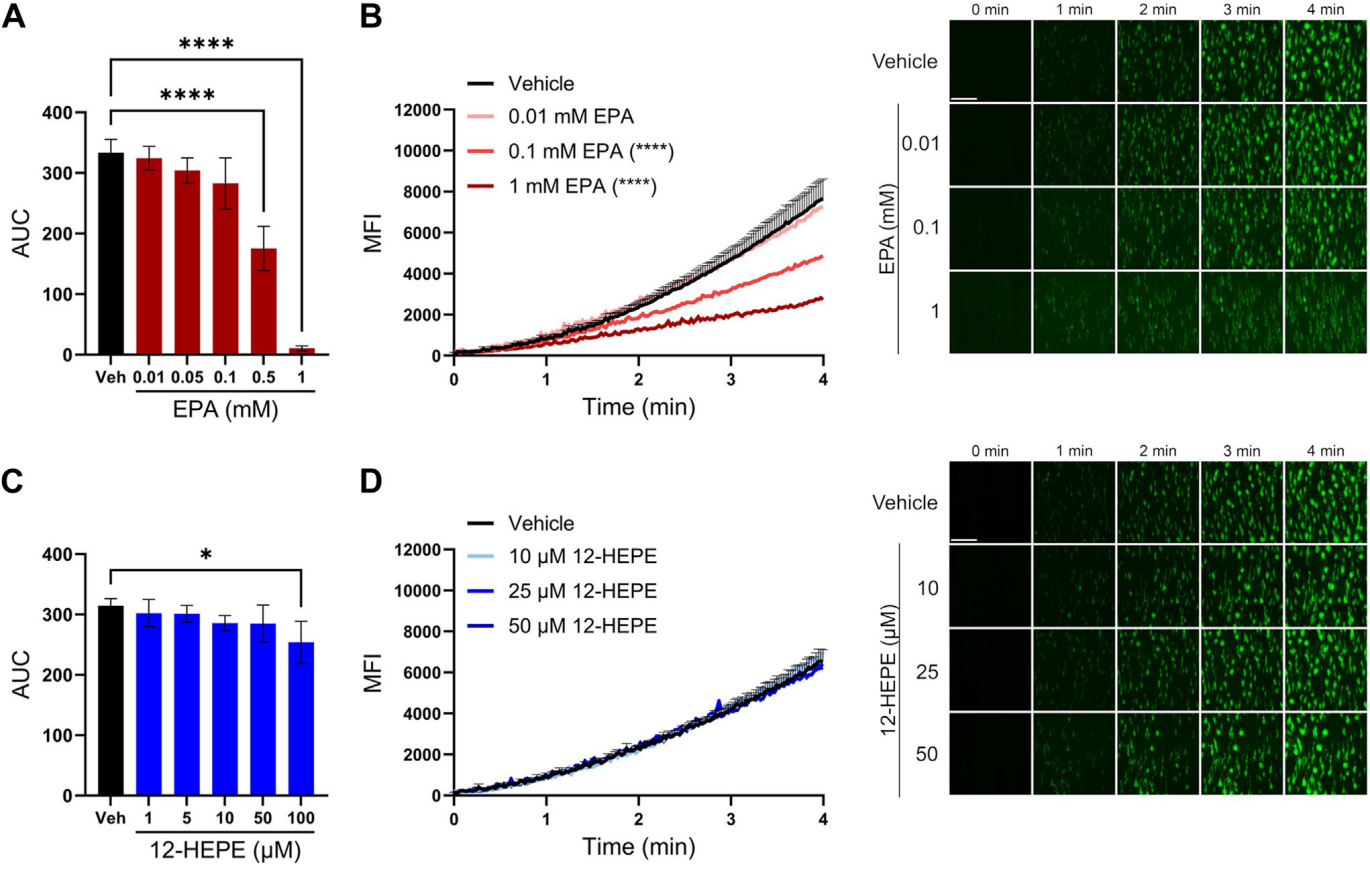
EPA attenuates ex vivo thrombus formation and platelet adhesion under arterial shear flow. Human whole blood treated with vehicle (DMSO), increasing concentrations of EPA (A), or 12-HEPE (C) for 10 min was perfused on a collagen-coated PL chip at arterial shear using T-TAS. Data are presented as mean area under the curve (AUC) ± SEM (n = 5). One-way ANOVA with Dunnett's multiple comparisons; ∗*P* < 0.05, ∗∗∗∗*P* < 0.0001. Quantification and representative images of sodium-citrated whole blood perfused through collage-coated chamber at atrial shear following incubation with vehicle (DMSO), increasing concentrations of EPA (B), or 12-HEPE (D) for 10 min. Scale bar represents 100 μm. Data are presented as mean MFI ± SEM (n = 5), and asterisks denote a statistical difference between vehicle determined via two-way ANOVA; ∗∗∗∗*P* < 0.0001.

To determine whether the inhibitory effects of EPA and 12-HEPE observed in washed platelets could be recapitulated in a more physiological setting of whole human blood under arterial flow, ex vivo thrombus formation and platelet adhesion were assessed using the T-TAS and microfluidic perfusion flow chamber assays, respectively. Whole blood was perfused over a collagen-coated PL chip with changes in pressure over time being analyzed via T-TAS (Fig. 5A, C). Similarly, sodium-citrated and recalcified whole blood was perfused though the microfluidic flow system over a collagen-coated chamber at arterial shear. Platelets were stained with DiOC_6_ for visualization (Fig. 5B, D). Platelet adhesion and accumulation to the collagen-coated surface were analyzed in samples incubated with EPA or 12-HEPE. Increasing concentrations of EPA exhibited a dose-dependent decrease in AUC in T-TAS, and 1 mM EPA fully inhibited ex vivo thrombus formation as assessed by T-TAS (Fig. 5A and supplemental Fig. S1A). Additionally, treatment with EPA was observed to significantly attenuate platelet adhesion to collagen under arterial flow at 0.1 mM (Fig. 5B). Whole blood treated with 12-HEPE decreased the AUC at 100 μM in T-TAS (Fig. 5C and supplemental Fig. S1B) but was not observed to alter platelet adhesion compared to vehicle in flow chamber conditions (Fig. 5D).

### 12-HEPE is produced in the presence of EPA in whole blood

To evaluate the ability of platelets to generate 12-HEPE in whole blood, samples were incubated with either vehicle (DMSO) or 500 μM EPA, followed by centrifugation to collect serum. Platelet counts across samples averaged (3.0 ± 0.4) × 10^8^ platelets/ml, within the physiological range. The concentration of EPA was selected based on the inhibition of ex vivo thrombus observed in the whole blood T-TAS assay and is consistent with plasma concentrations reported in prior clinical trials, which range from 124–326 μg/ml (409–1,077 μM) following EPA supplementation (16, 17, 34, 35). Oxylipin levels in the serum were quantified by LC/MS/MS or ELISA (Table 2). In the absence of an exogenous agonist, 12-HETE and TxB_2_ levels remained low. Incubation with EPA resulted in the generation of over 500 nM 12-HEPE. Additional EPA-derived oxylipins, including 15-HEPE and 5-HEPE, were also detected, albeit at lower concentrations, due to production by immune cells present in the whole blood (Table 2).

**Table 2:** Production of oxylipins in whole blood in the presence of EPA

Oxygenase	PUFA	Oxylipin	Oxylipin Production (nM)	
			Vehicle	500 μM EPA
COX-1	AA	TxB_2_	(3.5 ± 0.1)	(3.4 ± 0.1)
	EPA	TxB_3_	0	0
12-LOX	AA	12-HETE	(16.4 ± 3.5)	(16.4 ± 5.2)
	EPA	12-HEPE	0	(532.2 ± 109.3)
15-LOX	AA	15-HETE	0	0
	EPA	15-HEPE	0	(34.5 ± 6.7)
5-LOX	AA	5-HETE	0	0
	EPA	5-HEPE	0	(31.4 ± 4.4)

Serum was collected from whole blood incubated with vehicle (DMSO) or 500 μM EPA for 25 min and centrifuged at 2,000 *g* for 10 min. Oxylipins analyzed via LC/MS/MS or ELISA are presented as mean ± SEM (n = 4).

### EPA regulates platelet function independent of coagulation

**Fig. 6:**
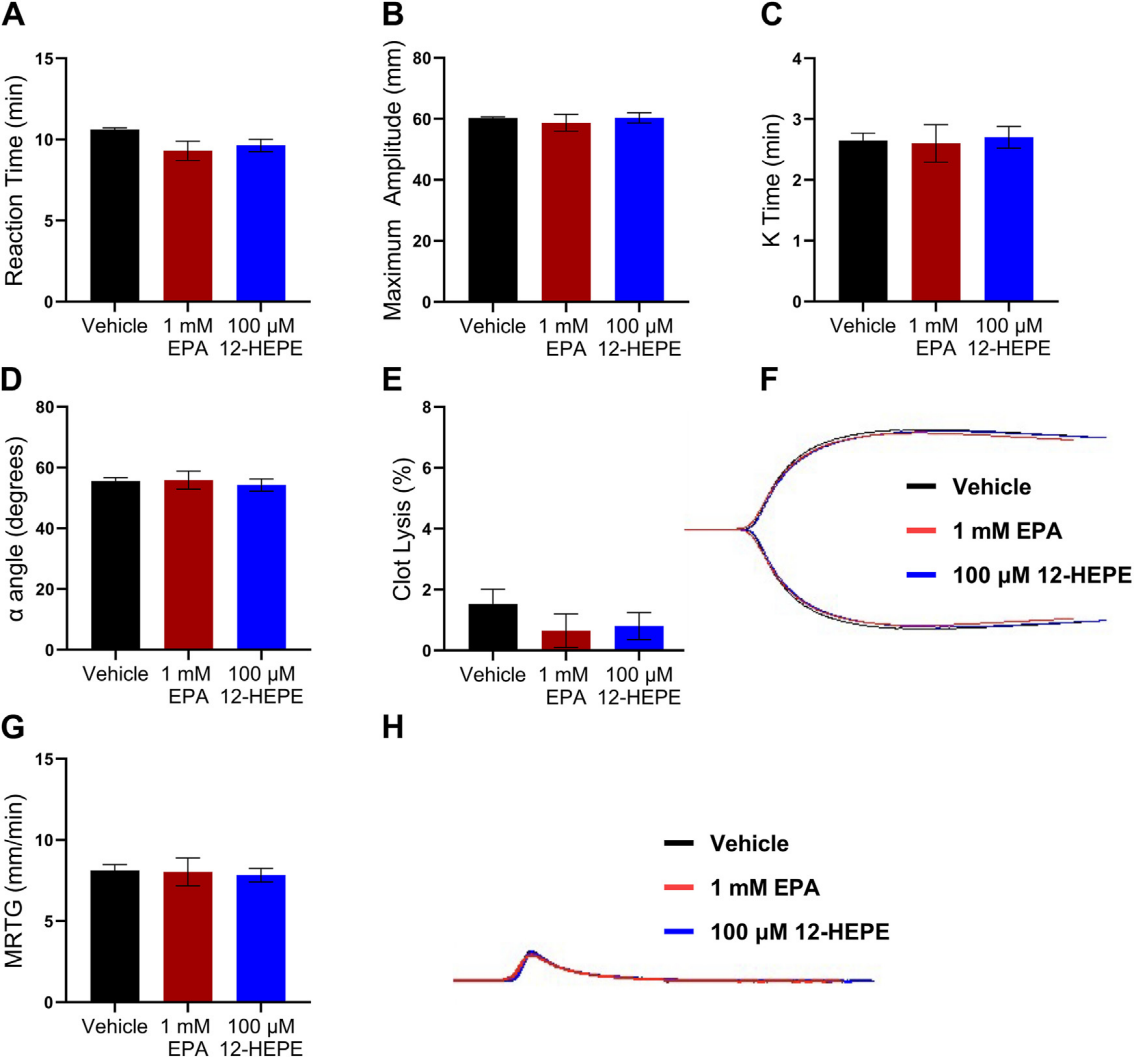
EPA and 12-HEPE do not alter coagulation parameters. Human whole blood was treated with vehicle (DMSO), 1 mM EPA, or 100 μM 12-HEPE for 10 min. Coagulation parameters were assessed using TEG (n = 4). The reaction time is the time to initial fibrin thread formation (A). Maximum amplitude represents clot strength (B). The time until the clot reaches a strength of 20 mm is the K time (C). The α angle is the rate of clot formation (D). Representative tracing of the coagulation parameters in panels A–E (F). Thrombin generation was calculated from velocity curves (G), and the representative tracing of the velocity curve graph clot strength over time used to calculation the rate of thrombin generation (H). One-way ANOVA with Dunnett's multiple comparisons.

To assess the potential of EPA and 12-HEPE in altering coagulation in the blood, several coagulation parameters were measured in human whole blood treated with the EPA or 12-HEPE using thromboelastography (TEG). By activating the contact coagulation pathway, the TEG analyzes the viscoelastic properties of whole blood clot formation under low shear stress. Concentrations of EPA (1 mM) and 12-HEPE (100 μM) were chosen based on the inhibition of thrombus formation observed in the whole blood assay of T-TAS. No changes in the time progression of clot formation were observed in the TEG; both reaction time (Fig. 6A) and K time (Fig. 6C) were consistent across all conditions. Similarly, no differences in clot development or clot strength were observed in α-angle (Fig. 6D), maximum amplitude (Fig. 6B), or maximum rate of thrombin generation (Fig. 6G). Clot stability was not altered by EPA or 12-HEPE, as no changes in clot lysis were observed (Fig. 6E). Representative tracings show no effect of EPA or 12-HEPE on coagulation parameters or thrombin formation (Fig. 6F, H).

## Discussion

ω-3 PUFAs are well-known for their numerous health benefits, specifically their ability to reduce the risk of cardiovascular events (36). Further, numerous studies have shown that an increased intake of ω-3 PUFAs through food or supplementation improves cardiovascular outcomes (3, 7, 17), which has led to the FDA approval of ω-3 fatty acid products for the prevention of cardiovascular disease (5, 37). Despite the wide use of over-the-counter fish oil supplements and FDA-approved products, the underlying mechanisms regulating the cardiovascular benefits of ω-3 PUFAs remain unknown. Previous mechanistic studies have focused on the ability of ω-3 PUFAs to lower triglyceride levels (5, 11). However, the benefits of ω-3 fatty acids appear to exceed their lipid-lowering effects in clinical trials, suggesting additional mechanistic studies are required (38).

While the mechanism by which ω-3 PUFAs alter platelet activation is not fully delineated, prior studies have demonstrated that supplementation with fish oil increases ω-3 PUFA content in the platelet membrane and attenuates platelet activation (12). These findings suggest that ω-3 PUFAs contribute to cardiovascular protection, at least in part, through their antiplatelet effects. EPA is the primary ω-3 PUFA in over-the-counter fish oil supplements (14), but the ability of EPA to alter platelet function remained unclear. Here, EPA is shown to attenuate platelet aggregation when stimulated through either GPVI or protease-activated receptors (PAR) (Fig. 1). Additionally, EPA regulates several key aspects of platelet activation, including integrin α_IIb_β_3_ activation, granule secretion (Fig. 3), and platelet adhesion (Fig. 5). EPA inhibits platelet activation in a dose-dependent manner, with effects observed at concentrations below 40 μM in washed platelets and below 500 μM in whole blood assays. While these levels of EPA may seem supraphysiologic, clinical trials have reported plasma concentrations of EPA between 124–326 μg/ml (409–1,077 μM) following supplementation (16, 17, 34, 35). Therefore, it is reasonable to suggest that EPA exhibits antiplatelet effects and reduces the risk of thrombosis in patients taking supplements containing high levels of EPA.

Previously, 12-LOX-derived oxylipins have been observed to play a critical role in platelet function (21, 22, 23). Studies have shown 12-LOX oxidizes AA, the most abundant membrane PUFA, to produce the prothrombotic oxylipin, 12-HETE (20, 32, 39). 12-LOX also oxidizes EPA with a comparable rate as that of AA (20). Without PUFA supplementation or changes to the platelet membrane, the absence or inhibition of 12-LOX suppresses platelet activation and thrombus formation by preventing 12-HETE production (2, 40, 41, 42). However, supplementation with PUFAs increases the abundance of non-AA PUFAs in the platelet membrane (12, 22). When these inhibitory PUFAs are enriched in the platelet membrane, the absence of 12-LOX prevents the ability of the PUFAs to inhibit platelet activation and thrombus formation (23). This suggests the role of 12-LOX in platelet activation and thrombosis depends on substrate availability. Hence, patients taking supplements containing elevated levels of EPA will have increased levels of EPA in the platelet membrane, resulting in increased production of 12-HEPE.

While several oxygenases are present in platelets, in vitro incubation with 10 μM EPA in washed platelets and 500 μM EPA in whole blood resulted in 12-HEPE being the most abundant oxylipin produced (Tables 1 and 2), as 12-LOX is highly expressed in the human platelet (40, 43). The generation of the 15-LOX product, 15-HEPE, is also increased in the presence of EPA (Fig. 2) but is less abundant compared to 12-HEPE. Platelets have been shown to only express low levels of 15-LOX-1 (44); therefore, the generation of the 15-LOX product could be produced by utilizing alternative pathways, such as CYP450s (18). As expected, the COX-1-derived metabolite of EPA (TxB_3_) was undetected (Table 1), as TxB_3_ production is minimal compared to production of HEPE products from exogenous EPA (45). Additionally, EPA is a poor substrate for COX-1, and several studies have reported that COX-1 converts EPA at about one-tenth the rate of AA in platelets, whereas the 12-LOX conversion of EPA is more efficient (32, 39, 46, 47). Previous clinical studies showed dietary ω-3 PUFAs decrease the formation of TxB_2,_ the AA-derived COX-1 oxylipin (48, 49). This led to the hypothesis that EPA competes with AA for COX-1 and 12-LOX binding, and the antiplatelet effects of EPA are realized by reducing the formation of prothrombotic AA-derived oxylipins, TxB_2,_ and 12-HETE. Interestingly, in our study, EPA did not alter the production of 12-HETE or TxB_2_ in intact platelets stimulated with collagen, suggesting that under these conditions, EPA does not interfere with the ability of 12-LOX or COX-1 to metabolize AA (Fig. 2B). However, upon thrombin stimulation, TxB_2_ production was reduced in the presence of EPA (Fig. 2D), although the decrease was modest compared to the robust increase in 12-HEPE. These results suggest the antiplatelet effects of EPA are not dependent on decreasing AA-derived oxylipin formation but are realized by increasing 12-HEPE formation.

The effects of 12-HEPE on platelet activation have remained controversial (20, 50), although this may be due, in part, to the agonists used in previous studies. Here, we not only demonstrate that 12-HEPE exhibits increased potency compared to EPA in washed platelets but also that 12-HEPE dose-dependently attenuates platelet aggregation, integrin activation, and granule secretion (Figs. 1 and 3). Interestingly, in PRP and whole blood conditions, 12-HEPE did not have as significant inhibitory effects compared to EPA. One potential limitation of the current study is the inability to match the concentrations of 12-HEPE and EPA in whole blood. Alternatively, the antiplatelet effects of 12-HEPE may be limited in the whole blood assays used in the current study due to binding by albumin or instability of the oxylipin. Despite these limitations, we demonstrated that EPA significantly enhances platelet production of 12-HEPE in whole blood (Table 2), and EPA notably reduces platelet adhesion and thrombus formation in whole blood (Fig. 5). Therefore, the antiplatelet effects of EPA may be regulated by local intracellular formation of 12-HEPE within the platelet. Previous studies have shown that 12-HEPE activates intracellular peroxisome proliferator-activated (PPAR) receptors (51, 52), a mechanism through which other 12-LOX oxylipins exhibit their antiplatelet effects (22).

Bleeding risk is a significant clinical concern with any endogenous oxylipin or exogenous compound that alters platelet function. Bleeding events have been observed with many antiplatelet agents currently in use (53), as well as in the REDUCE-IT trial, which showed supplementation was associated with a small increase in bleeding risk (17). To identify whether EPA or 12-HEPE alters coagulation in the blood, TEG was used to analyze coagulation parameters following incubation with EPA or 12-HEPE. Neither EPA nor 12-HEPE was found to alter coagulation parameters, suggesting bleeding may not be a risk factor with elevated EPA or formation of 12-HEPE in the platelet; however, future studies are required to determine the effects of long-term supplementation on coagulation parameters and bleeding risk due to EPA or its oxylipins.

**Fig. 7:**
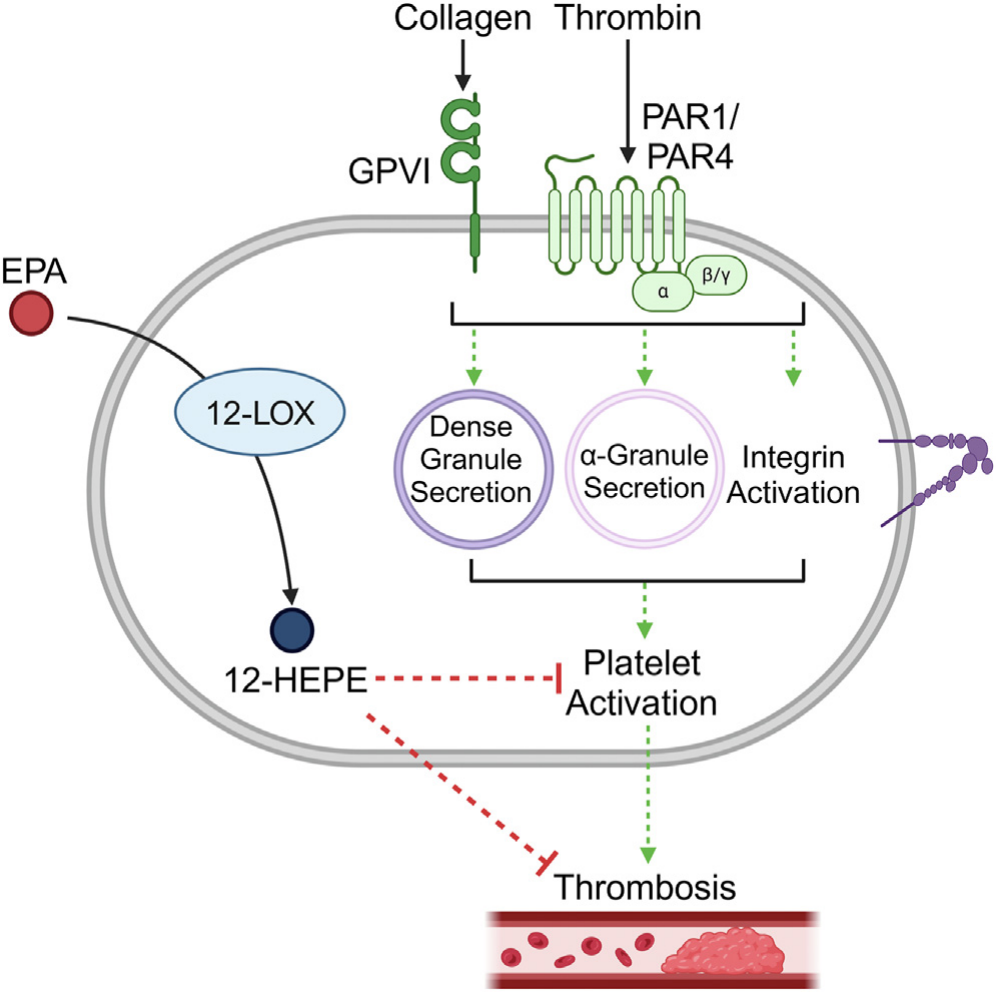
Proposed model of 12-HEPE inhibitory regulation of platelet function and clot formation. Schematic overview of the underlying inhibitory effect of the EPA bioactive metabolite, 12-HEPE. Within platelets, 12-lipoxygenase (12-LOX) metabolizes free EPA into the bioactive lipid, 12-HEPE. 12-HEPE inhibits dense granule secretion, α-granule secretion, and integrin activation, leading to inhibition of platelet aggregation in response to collagen. Created in BioRender. https://BioRender.com/b11j269.

12-HEPE formation by the platelet is increased in the presence of EPA, and both EPA and 12-HEPE exhibit potent antiplatelet effects. The data presented in this study support the hypothesis that the cardiovascular protective effects realized in individuals taking dietary supplements containing high levels of EPA are likely in part regulated by 12-HEPE (Fig. 7). Although much work remains to fully understand the role of 12-LOX in regulating in vivo clot formation following EPA supplementation and the mechanisms by which 12-HEPE inhibits platelet activation, the work presented here is a significant advancement in the field. This study delineates, for the first time, the potential underlying mechanism by which EPA regulates human platelet activation. Specifically, EPA inhibits platelet activation and clot formation, offering protective effects that are independent of its well-established role in lowering blood triglyceride levels. These findings offer new insights into how EPA confers cardiovascular benefits, emphasizing its potential as a therapeutic intervention for atherothrombotic diseases.

## Data availability

All data will be shared upon reasonable request by contacting the corresponding author, Michael Holinstat (University of Michigan Medical School; mholinst@med.umich.edu).

## Supplemental data

This article contains supplemental data.

## Conflict of interest

The authors declare the following financial interests/personal relationships which may be considered as potential competing interests: M. H. is a consultant for Veralox Therapeutics, Cereno Scientific, and Lexicon Pharmaceuticals. M. H. is an equity holder and holds patents that have been licensed by Veralox Therapeutics and Cereno Scientific. T. H. holds patents that have been licensed by Veralox Therapeutics and Cereno Scientific. All other authors declare no competing interests for the work reported in this manuscript.
