# Supplementary material for: The EPA oxylipin, 12-HEPE, directly regulates human platelet activity
Source: J Lipid Res. 2025 Apr 16;66(5):100807. doi: 10.1016/j.jlr.2025.100807 (PMC12144432; doi:10.1016/j.jlr.2025.100807)
Supplement: Supplementary Materials [file mmc1.pdf]

SUPPLEMENTAL FIGURES

The EPA oxylipin, 12-HEPE, directly regulates human platelet activity

Krista Goerger[1], Livia Stanger[1], Andrew Rickenberg[1], Anthony Nguyễn[2], Taekyu Lee[1], Theodore R. Holman[2], Michael Holinstat[1,3]

[1]Department of Pharmacology, University of Michigan Medical School, Ann Arbor, MI

[2]Department of Chemistry and Biochemistry, University of Santa Cruz, Santa Cruz, CA

[3]Department of Internal Medicine, University of Michigan Medical School, Ann Arbor, MI

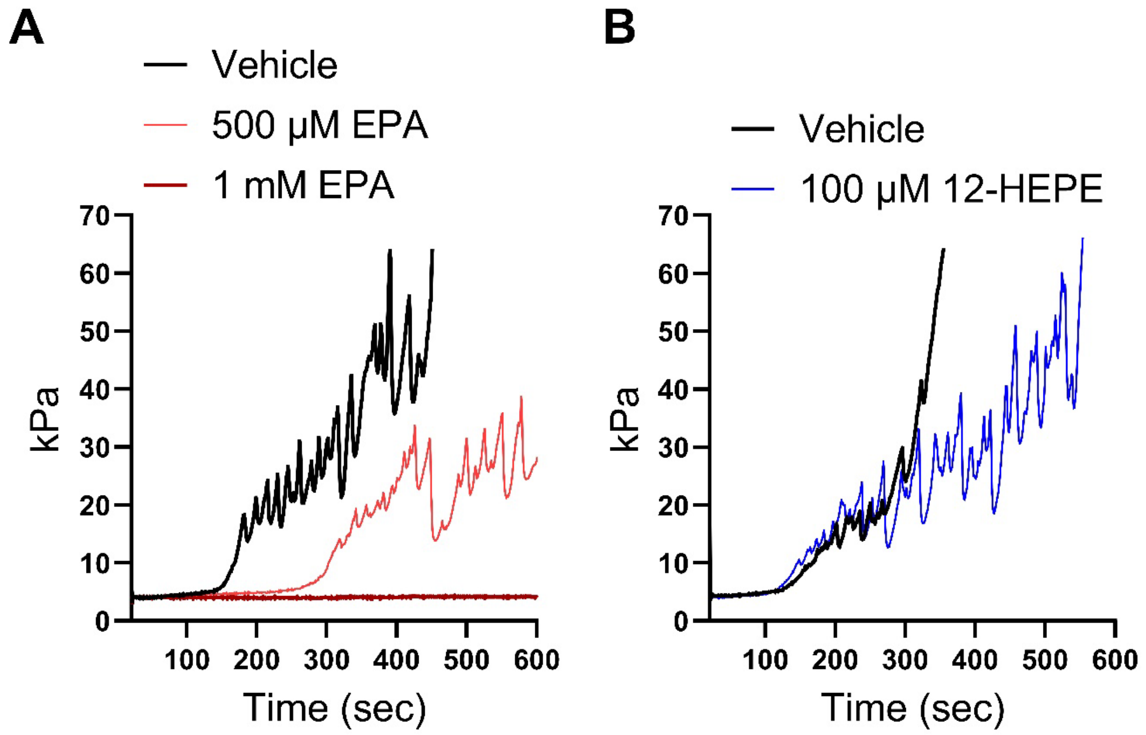

Supplemental Figure S1: Representative traces of thrombus formation assessed via Total-Thrombus Analysis System (T-TAS) displayed in **Figures 5A & C**. Human whole blood was treated with EPA and 12-HEPE for 10 minutes at 37°C prior to thrombus analysis.
